# CARDIOVASCULAR RISK BEFORE AND AFTER SURGICAL TREATMENT OF SEVERE OBESITY

**DOI:** 10.1590/0102-6720202400066e1860

**Published:** 2025-01-27

**Authors:** Lilian CARDIA, Alexandre Viera GADDUCCI, Denis PAJECKI, Marco Aurelio SANTO, Roberto DE CLEVA

**Affiliations:** 1 Universidade de São Paulo, Faculty of Medicine, Department of Gastroenterology – São Paulo (SP), Brazil.

**Keywords:** Obesity, Bariatric Surgery, Insulin Resistance, Metabolic Syndrome, Lipoproteins, Obesidade, Cirurgia Bariátrica, Resistência à Insulina, Síndrome Metabólica, Lipoproteínas

## Abstract

**BACKGROUND:**

Obesity is a predisposing factor for serious comorbidities, particularly those related to elevated cardiovascular mortality. The atherogenic index of plasma (AIP) has been shown to be a useful indicator of patients with insulin resistance.

**AIMS:**

The aim of this study was to assess cardiovascular risk before and after surgical treatment of obesity.

**METHODS:**

A total of 615 patients undergoing bariatric surgery between 2007 and 2012 were evaluated using the analysis of electronic records (triglyceride/high-density lipoprotein cholesterol) before and after surgery. The AIP levels >3.5 mg/dL for men and >2.5 mg/dL for women were insulin-resistant and predisposed to cardiovascular events.

**RESULTS:**

A total of 117 men had an AIP >3.5 mg/dL during the preoperative period, 13.5% during the early postoperative period, 14.3% during the intermediate period, and 18.2% during the late postoperative period. Among 498 women, 56.2% had an AIP >2.5 mg/dL before surgery, 17.9% in early postoperative period, 13.5% in the intermediate period, and 11.4% in the late period.

**CONCLUSIONS:**

Bariatric surgery resulted in a significant effect on the AIP, insulin resistance, metabolic syndrome, and therefore, the risk of cardiovascular diseases.

## RESUMO

**RACIONAL:**

A obesidade é fator predisponente para patologias potencialmente graves, destacando-se as relacionadas a uma maior mortalidade cardiovascular. O índice aterogênico plasmático (TG/HDL-c) revelou ser um indicador prático, não invasivo e com boa acurácia para identificar pacientes com resistência à insulina, principal componente da síndrome metabólica, além de preditor independente para o desenvolvimento de doenças cardiovasculares.

**OBJETIVOS:**

Avaliar risco cardiovascular antes e após o tratamento cirúrgico da obesidade.

**MÉTODOS:**

Foram estudados 615 pacientes submetidos à cirurgia bariátrica, no período de 2007 a 2012, através da análise dos registros eletrônicos (triglicérides, HDL-c. LDL-c e colesterol total) antes e após a cirurgia. Aqueles que apresentaram índice aterogênico plasmático >3,5 mg/dl e 2,5 mg/dl, respectivamente, para homens e mulheres, foram considerados resistentes à insulina e com alto risco de doença cardiovascular.

**RESULTADOS:**

Entre 117 homens, 53% apresentaram índice aterogênico plasmático >3,5 mg/dl no período pré-operatório, 13,5% no pós-operatório precoce, 14,3% no intermediário e 18,2% no tardio. Entre 498 mulheres, 56,2% apresentaram índice aterogênico plasmático >2,5 mg/dl no pré-operatório, 17,9% no pós-operatório precoce, 13,5% no intermediário e 11,4% no tardio.

**CONCLUSÕES:**

A intervenção cirúrgica para o tratamento da obesidade promoveu impacto significativo na relação TG/HDL-c, na resistência à insulina, na síndrome metabólica e, consequentemente, no risco de doenças cardiovasculares.

## INTRODUCTION

According to the World Health Organization, obesity and overweight are among the largest epidemics of the century. It is estimated that around 2 billion adults around the world are overweight, and beyond 700 million have obesity^27^. The National Health Survey (PNS, 2020) in Brazil confirms the prevalence of obesity among women (29.5%) and men (21.8%)^4^.

Obesity is a complex and multifactorial disease characterized by excessive accumulation of fat with potentially serious comorbidities. It exhibits a wide interaction between genetic and environmental factors with significant psychological and social dimensions^18^.

Several comorbidities are associated with obesity, especially those related to higher cardiovascular mortality, such as systemic arterial hypertension, type II diabetes mellitus, dyslipidemia, and metabolic syndrome (MetS). These conditions have a direct impact on morbidity and mortality^5,12^.

Clinical treatments of severe obesity had little effectiveness, with a recurrence of up to 95%^25^. For these patients, bariatric and metabolic surgery is considered the treatment of choice due to significant weight loss and remission of associated diseases, reducing the risk of mortality and improving quality of life^7^.

Insulin resistance (IR) is one of the main components of MetS and an independent predictor of cardiovascular diseases (CVDs)^17^, hepatic steatosis, and steatohepatitis (metabolic associated steatotic liver disease)^21^. IR is related to the degree of obesity, with improvement observed after weight loss^10^. High levels of triglycerides (TG) and a decrease in high-density lipoprotein cholesterol (HDL-c) are frequent consequences of IR^3^.

The atherogenic index of plasma (AIP — the TG/HDL-c ratio) identifies patients with IR and MetS^8,14,23^. A TG/HDL-c ratio >3.5 mg/dL has been considered ideal for identifying insulin-dependent patients with a sensitivity and specificity comparable to the criteria proposed for diagnosing MetS^16^. Age and gender factors are equally important and should be considered when using the TG/HDL-c ratio to assess cardiovascular risk^26^. Salazar et al. suggest that men and women with a TG/HDL-c ratio higher than 3.5 and 2.5 mg/dL, respectively, had the worst cardiovascular outcomes^20^.

The aim of this study was to assess cardiovascular risk before and after surgical treatment of severe obesity.

## METHODS

The retrospective study evaluated electronic records of 615 individuals who underwent metabolic surgery in a Bariatric and Metabolic Surgery Unit of the University of Sao Paulo Medical School between 2007 and 2012. TG and HDL-c data were collected from the following periods: preoperative (PREOP), early postoperative (EARLY PO), between 6 and 12 months, intermediate postoperative (INTER PO), between 12 and 24 months, and late postoperative (LATE PO), between 24 and 36 months after surgery.

The TG/HDL-c ratio (AIP) was calculated from the plasma TG and HDL-c values (in mg/dL) for each period. The AIP was analyzed based on gender ^26^. Men and women with an AIP >3.5 and 2.5 mg/dL, respectively, were considered insulin-resistant and at high risk of developing CVD.

The research was approved by the Research Ethics Committee of the University of Sao Paulo Medical School (number 03006112.6.0000.0068).

Statistics were conducted using Statistical Package for the Social Sciences (SPSS) 12 (SPSS, Chicago, Illinois). For continuous variables, the data were presented as means and standard deviation, while for categorical variables, they were presented as percentages. The Mc-Nemar test was utilized to evaluate the comparison between the periods examined. The statistical significance was determined to be 5% (p<0.05).

## RESULTS

The AIP was calculated for 615 patients with severe obesity before bariatric surgery (Table 1). The mean TG/HDL was 3.6±2.7 in the PREOP, 1.94±1.3 in the EARLY PO, 1.8±1.5 in the INTER PO and 1.7±1.2 in the LATE PO (Table 1). Of the 117 men evaluated in the PREOP, 62 patients (53%) had AIP >3.5 mg/dL (mean 7.1±3.7), indicating the presence of IR and MetS. Of the 52 patients studied in the EARLY PO, seven patients (13.5%) had AIP >3.5 mg/dL (mean 6.0±1.5). In the INTER PO, among 35 patients evaluated, five patients (14.3%) had AIP >3.5 mg/dL (mean 5.7±2.3). In the LATE PO, among 22 patients evaluated, four patients (18.2%) had AIP >3.5 mg/dL (mean 6.4±1.8). There was a significant decrease in the number of patients with MetS and a high risk of CVD between the PREOP and EARLY PO periods (p<0.001), between the PREOP and INTER PO periods (p<0.001), and between the PREOP and LATE PO periods (p=0.016).

**Table 1:** Atherogenic index of plasma before and after surgical treatment for obesity.

	PREOP			EARLY PO				INTER PO				LATE PO			
	Md	%	95%CI	Md	%	95%CI	p-value	Md	%	95%CI	p-value	Md	%	95%CI	p-value
Male (n)	117			52				35				22			
TG/HDL-c ≥3.5	7.1±3.7	53.0	44.0–61.8	6.0±1.5	13.5	6.5–25.7	<0.001	5.7±2.3	14.3	5.9–30.0	<0.001	6.4±0.5	18.2	6.9–39.3	0.016
TG/HDL-c ≤3.5	2.4±0.7			1.6±0.7				1.6±0.8				1.2±0.5			
Female (n)	498			263				192				114			
TG/HDL-c ≥2.5	4.5±2.1	56.2	51.8–60.5	3.8±1.7	17.9	13.7–23.0	<0.001	4.3±2.5	13.5	9.4–19.2	<0.001	3.6±1.2	11.4	6.7–18.7	<0.001
TG/HDL-c ≤2.5	1.7±0.5			1.5±0.5				1.3±0.5				1.4±0.5			

Results are expressed as mean and SD. PREOP: preoperative period; EARLY PO: early postoperative period; INTER PO: intermediate postoperative period; LATE PO: late postoperative period; CI: confidence interval; Md: median.

Of the 498 women evaluated in PREOP, 280 patients (56.2%) had AIP >2.5 mg/dL (mean 4.5±2.1). During the EARLY PO, among 263 patients evaluated, 47 patients (17.9%) had AIP >2.5 mg/dL (mean 3.8±1.7), resulting in a significant decrease in MetS and high CVD risk. Of the 192 patients evaluated during INTER PO, 26 patients (13.5%) had AIP >2.5 mg/dL (mean 4.3±2.5). Among 114 patients evaluated in the LATE PO, 13 patients (11.4%) had AIP >2.5 mg/dL (mean 3.6±1.2). There was a significant decrease in the number of patients with MetS and a high risk of CVD between the PREOP and EARLY PO periods (p<0.001), between the PREOP and INTER PO periods (p<0.001), and between the PREOP and LATE PO periods (p<0.001).

**Figure 1:**
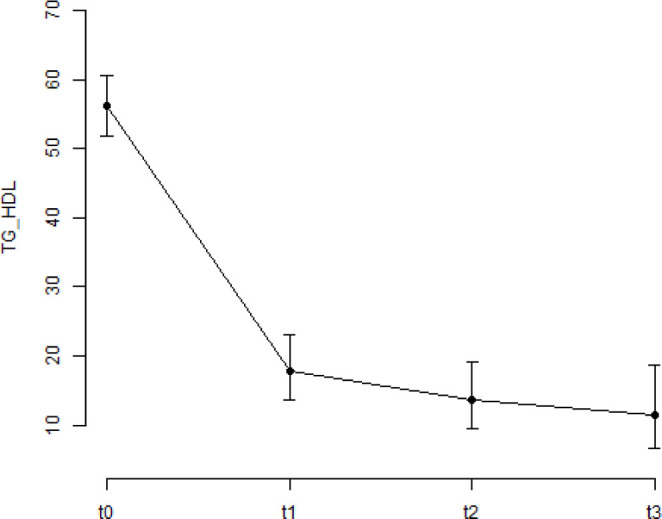
Percentage of male patients with atherogenic index of plasma >3.5 mg/dL in preoperative, early postoperative, intermediate postoperative, and late postoperative periods. The bars represent the confidence interval (95% CI).

**Figure 2:**
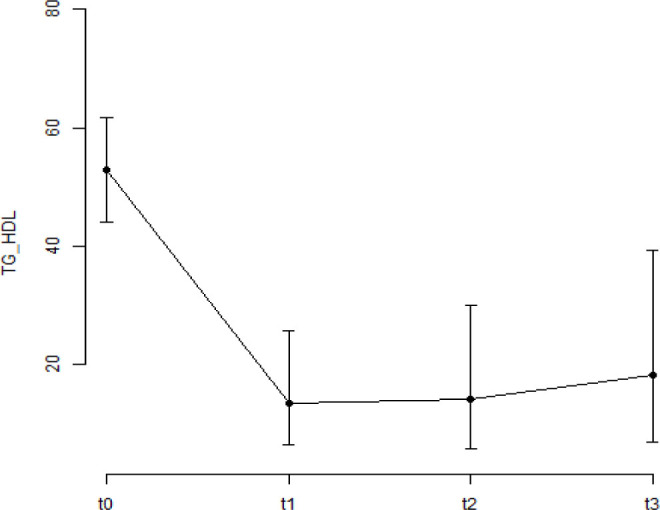
Percentage of female patients with an atherogenic index of plasma >2.5 mg/dL in preoperative, early postoperative, intermediate postoperative, and late postoperative periods. The bars represent the confidence interval (95% CI). The bars represent the confidence interval (95% CI).

The percentage evaluation of patients during the surgical periods is shown in Figures 1 and 2.

TG/HDL: triglyceride/high-density lipoprotein cholesterol.

TG/HDL: triglyceride/high-density lipoprotein cholesterol.

## DISCUSSION

Metabolic alterations, such as IR and hyperinsulinemia, can lead to changes in TG and total cholesterol. These changes can predispose to atherosclerotic disease and an increased risk of cardiovascular events. Studies indicate that the AIP, determined by routine tests, has a strong correlation with cardiovascular risk and acute myocardial infarction regardless of gender^11,13,15^.

The relationship with the development of atherosclerosis from high concentrations of lipoproteins results in an increase in LDL-c^19^. It has been demonstrated that AIP is an indicator of the progression and severity of coronary lesions^6^. AIP is also important for identifying patients with IR and MetS. Elevated TG and reduced HDL-c levels are independent risk factors for coronary heart disease. Additionally, these alterations are observed in the presence of IR^9^.

Recent evidence suggests that obesity-associated coronary heart disease may be a direct outcome of excessive fat and visceral adiposity, through mechanisms involving a low-grade inflammatory state, endothelial dysfunction, and regulation of pro-inflammatory cytokines^2^.

Our study shows that individuals with obesity in the preoperative period had a high AIP (53% of men and 56.2% of women), demonstrating the high prevalence of IR, MetS, and increased cardiovascular risk in this population.

Nevertheless, we can observe a notable decrease in IR, MetS, and cardiovascular risk assessed at other times during the analysis, which persists for 36 months after surgical treatment for obesity.

The percentage decrease is similar between men (53% in PREOP, 13.5% in EARLY PO, 14.3% in INTER PO, and 18.2% in LATE PO) and women (56% in PREOP, 17.9% in EARLY PO, 13.5% in INTER PO, and 11.4% in LATE PO), showing the effectiveness of bariatric surgery in controlling dyslipidemia.

The weight loss induced by bariatric surgery is accompanied by a reduction in cardiovascular risk and mortality^1,22^. Vest et al. conducted an analysis of 73 studies involving 19,543 patients, including 76% women. The findings revealed an average reduction in excess weight of 54%, and improvement in hypertension, diabetes, and dyslipidemia in 44%, 24%, and 44% of patients, respectively, with an average follow-up of 57.8 months^24^.

Our research has some limitations. The current investigation is a retrospective study. Second, the presence of comorbidities such as hypertension, diabetes, and the use of medication for dyslipidemia were not included. Information on potential risk factors for CVD such as diet, physical activity, genetic factors, and demographic and anthropometric variables was not collected during our study. Another limitation is the decreasing number of patients in the late follow-up of bariatric surgery.

## CONCLUSIONS

Bariatric surgery was associated with a significant decrease in AIP, reducing cardiovascular risk in individuals with severe obesity. This reduction persists for at least 3 years after surgery.
